# Supplementary material for: Growth Trajectories in Genetic Subtypes of Prader–Willi Syndrome
Source: Genes (Basel). 2020 Jul 2;11(7):736. doi: 10.3390/genes11070736 (PMC7397071; doi:10.3390/genes11070736)
Supplement: Supplementary file 1 [file genes-11-00736-s001.pdf]

genes

MDPI

Supplementary data

Table S1. Comparison of the Akaike information criterion and Bayesian information criterion for a LMM (random slope only), a LMM (random slope and random intercept and a LMM with AR(1) correlation structure when fitting growth models for height for the female deletion subgroup.

Gender	Fitted Model	Deletion subgroup, Outcome: Height					
		4 knots (2,5,10,15)		3 knots (2,7,15)		2 knots (5,10)	
		AIC	*BIC*	*AIC*	*BIC*	*AIC*	*BIC*
	Random slope only	4876.0	4914.5	4888.2	4921.9	5112.5	5145.4
Female	Random slope and intercept	4488.5	4536.6	4513.8	4557.1	4882.9	4921.4
	Random effects + AR(1)	3746.3	3799.2	3747.9	3796.1	3918.8	3962.1

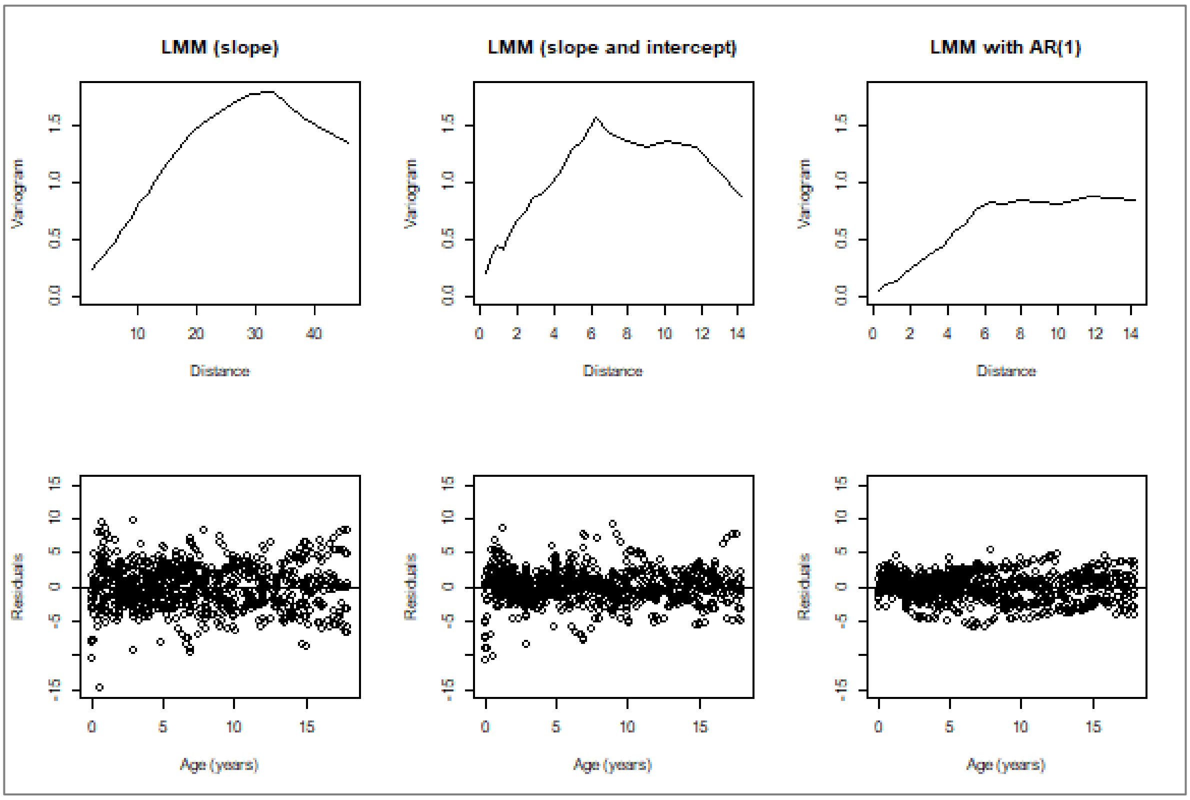

Figure S1. Comparison of the variograms and residual plots for a LMM (random slope only), a LMM (random slope and random intercept and a LMM with AR(1) correlation structure when fitting growth models (with 4 knots) for height for the female deletion subgroup.

Table S2. Mean difference in rate of growth across age intervals between individuals with the deletion and non-deletion mechanism of PWS, estimated using linear mixed models with linear splines (adjusted for year of birth), with an interaction term between age and genetic subtype.

Outcome	Age (years)	Average difference in mean rate of growth *(Deletion – Non-deletion)*					
		Females			Males		
		Estimate	*[95% CI]*	*P-value*	*Estimate*	*[95% CI]*	*P-value*
Height (cm/year)	<2	0.11	[-1.09, 1.30]	0.86	1.31	[-0.24, 2.87]	0.10
	2-5	0.40	[-1.20, 2.01]	0.62	-1.27	[-3.11, 0.58]	0.18
	5-10	0.15	[-0.92, 1.21]	0.79	-1.37	[-2.50, -0.24]	0.02
	10-15	-0.81	[-1.82, 0.20]	0.11	2.80	[1.68, 3.92]	0.01
	>15	1.04	[-0.81, 2.89]	0.27	1.90	[-0.20, 4.01]	0.08
Weight (kg/year)	<2	0.73	[-0.71, 2.17]	0.32	-0.18	[-2.61, 2.25]	0.89
	2-5	-0.40	[-2.18, 1.39]	0.66	0.24	[-2.03, 2.51]	0.84
	5-10	1.07	[-0.10, 2.24]	0.07	-0.23	[-1.67, 1.21]	0.76
	10-15	-1.59	[-2.69, -0.48]	0.01	3.71	[2.30, 5.12]	<0.01
	>15	-1.83	[-3.86, 0.21]	0.08	1.30	[-1.36, 3.96]	0.34
BMI ((kg/m^2)/ year)	<2	1.36	[0.57, 2.15]	<0.01	1.04	[-0.14, 2.22]	0.08
	2-5	-1.07	[-2.09, -0.05]	0.04	-0.57	[-1.85, 0.70]	0.38
	5-10	-0.09	[-0.76, 0.57]	0.78	-0.40	[-1.20, 0.40]	0.33
	10-15	-0.38	[-1.00, 0.24]	0.23	0.43	[-0.36, 1.21]	0.29
	>15	-0.93	[-2.10, 0.23]	0.12	0.16	[-1.31, 1.63]	0.83
